# Revealing the Mechanism of NLRP3 Inflammatory Pathway Activation through K^+^ Efflux Induced by PLO via Signal Point Mutations

**DOI:** 10.3390/ijms25126703

**Published:** 2024-06-18

**Authors:** Qiang Shan, Wenbo Ma, Bolin Li, Qian Li, Xue Wang, Yanan Li, Jiufeng Wang, Yaohong Zhu, Ning Liu

**Affiliations:** 1 College of Veterinary Medicine, Northeast Agricultural University, Harbin 150030, China; xiaoqiangdebaobao@163.com (Q.S.); mawenbozi@163.com (W.M.); eightyeighty180@hotmail.com (B.L.); l33980715@163.com (Q.L.); 13796685756@163.com (Y.L.); 2 College of Veterinary Medicine, China Agricultural University, Beijing 100093, China; ivywang0913@163.com (X.W.); jiufeng_wang@hotmail.com (J.W.); zhu_yaohong@hotmail.com (Y.Z.); 3 Key Laboratory of the Provincial Education Department of Heilongjiang for Common Animal Disease Prevention and Treatment, College of Veterinary Medicine, Northeast Agricultural University, Harbin 150030, China

**Keywords:** *Trueperella pyogenes*, PLO, cholesterol-dependent cytolysin, NLRP3, K^+^ efflux, single-point mutation

## Abstract

*Trueperella pyogenes* is an important opportunistic pathogenic bacterium widely distributed in the environment. Pyolysin (PLO) is a primary virulence factor of *T. pyogenes* and capable of lysing many different cells. PLO is a member of the cholesterol-dependent cytolysin (CDC) family of which the primary structure only presents a low level of homology with other members from 31% to 45%. By deeply studying PLO, we can understand the overall pathogenic mechanism of CDC family proteins. This study established a mouse muscle tissue model infected with recombinant PLO (rPLO) and its single-point mutations, rPLO N139K and rPLO F240A, and explored its mechanism of causing inflammatory damage. The inflammatory injury abilities of rPLO N139K and rPLO F240A are significantly reduced compared to rPLO. This study elaborated on the inflammatory mechanism of PLO by examining its unit point mutations in detail. Our data also provide a theoretical basis and practical significance for future research on toxins and bacteria.

## 1. Introduction

*Trueperella pyogenes* is an important opportunistic pathogenic bacterium widely distributed in the environment [1]. Under certain conditions, it can develop into pathogenic bacteria infecting livestock, wildlife, community animals, and humans and leading to purulent infections of tissues and organ mucosa [2]. *T. pyogenes* infection often leads to severe inflammatory diseases in animals, such as mastitis, abscess, pneumonia, and lymphadenitis [3]. *T. pyogenes* produces several known and putative virulence factors, among which purulent pyolysin (PLO) is the only hemolysin expressed in this organism and has been reported to make significant contributions to its pathogenicity [4,5].

PLO is a primary virulence factor of *T. pyogenes* and capable of lysing immune cells [6]. PLO is a member of the cholesterol-dependent cytolysin (CDC) family of which the primary structure only presents a low level of homology with other members from 31% to 45% [7]. Such low homology can still maintain the special structure and function of the CDC family in PLO. Some studies have shown that the pathogenicity of purulent *T. pyogenes* strains using deficient PLO or mutated PLO is reduced compared to strains that produce the same gene toxin in mice [3,8]. Our previous study indicated that intramuscular administration of recombinant PLO (rPLO) protein can upregulate the expression of pro-inflammatory cytokine interleukin-1β (IL-1β) and IL-18 in mice and cause severe tissue damage [7]. Moreover, we found that PLO is lethal to mice.

Many common inflammatory diseases can activate the cytoplasmic innate immune signaling receptor NLRP3 (NOD-, LRR-, and pyrin-domain 3) [9]. Once activated, NLRP3 will cause the assembly of inflammasomes into nuclei, leading to caspase-1-mediated proteolytic activation of the IL-1β [10]. The protein hydrolysis activation of family cytokines induces inflammatory and pyrolytic cell death [9]. The first known NLRP3 activators are those that open pores or channels in the plasma membrane or ion carriers that can make the membrane potassium-permeable [9]. If there are dying cells near cells containing inflammasomes, high levels of extracellular ATP will appear, which will also increase the probability of opening the P2X purine receptor 7 (P2X7) channel [11], leading to ion flux. Once opened, this channel allows potassium ions to flow out of the cell, achieving ion balance through the influx of calcium ions. Other bacterial pore-forming toxins, including perforin O and streptolysin O, also activate this pathway by allowing potassium ions to efflux [12].

Through in-depth research on PLO, we can understand the overall pathogenic mechanism of CDC family proteins. This study established a mouse muscle tissue model infected with rPLO by performing single-point mutations and explored its mechanism of causing inflammatory damage. The inflammatory injury abilities of these mutants are significantly reduced compared to rPLO. This lays the foundation for understanding the pathogenicity of the entire CDC family and preventing pathogenic bacteria that produce CDC proteins in the future.

## 2. Results

### 2.1. Mutation, Expression, and Purification of the PLO Protein

This study successfully constructed two rPLO mutants with single-point mutations (rPLO N139K and rPLO F240A). During the expression and purification processes, the rPLO mutants were prepared using the same processes as rPLO, and no significant difference in solubility was detected between the mutants and rPLO. By comparing the sequences of the *plo* gene with other CDC family members, it was found that the sequence identity between the *plo* gene and other CDC family members was only 31–45%, while the identity between other CDC family members was between 40% and 70% [7]. Such low sequence identity can also maintain the characteristics of the CDC family in PLO, indicating that the conserved sequences of PLO and other CDC toxins are crucial for PLO to exercise its hemolytic and pore-forming functions. Our previous studies have shown that sites 139 and 240 are highly conserved compared to other pore-forming toxins [7]. Mutations at conserved sites 139 and 240 can fully demonstrate the functional and structural relationships of PLO.

**Figure 1:**
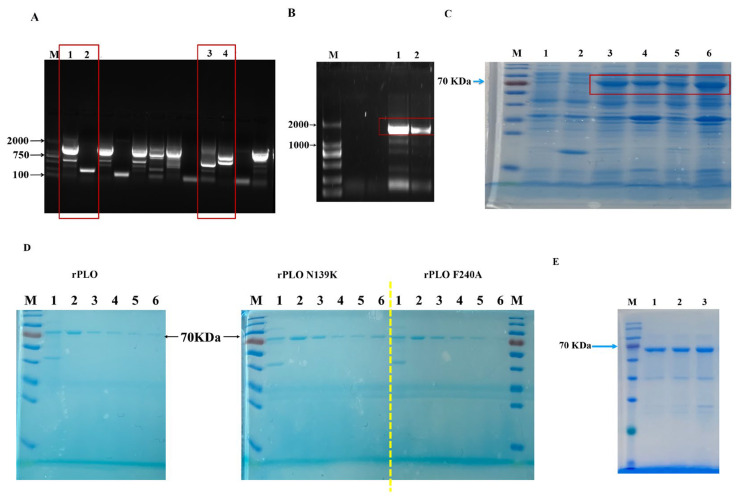
In vitro recombinant PLO expression process. (**A**) Two fragments of the same mutation were subjected to PCR and nucleic acid electrophoresis. M: Marker; 1: rPLO-139-1; 2: rPLO-139-2; 3: rPLO240-1; 4: rPLO-240-2; (**B**) Two fragments of the mutation were fused into a complete sequence. M: Marker; 1: rPLO-139; 2: rPLO-240; (**C**) Proteins were expressed in the supernatant and pellet, respectively. M: Marker; 1: Competent cells without expression; 2: Empty plasmid; 3: rPLO supernatant; 4: rPLO precipitate; 5: rPLO-139 supernatant; 6: rPLO-139 precipitate; (**D**) Different gradients of imidazole eluted protein concentrations. M: Marker; 1–6: 50~300 mM imidazole; (**E**) Identification of purified protein with SDS-PAGE M: Marker; 1: rPLO; 2: rPLO-139; 3: rPLO-240.

This study used fusion PCR technology to perform PCR on the front and back sequences of the mutated sites. The results of the two-stage PCR are shown in Figure 1A. After selecting the correct position of the band for DNA recovery and continuing with the PCR reaction, the complete *plo* gene sequence after mutation can be obtained (Figure 1B). The correct size of the *plo* gene indicates that the two sequences were connected together. It was found that rPLO and its mutants were expressed in both the supernatant and the precipitate (Figure 1C). Proteins from the supernatant were selected for subsequent experiments in this study. Different concentrations of imidazole solutions (50–300 mM) were used to elute the proteins from the adsorption column. The protein concentrations eluted by 50~200 mM imidazole solutions were higher than imidazole solutions of other concentrations. After eluting by 50–200 mM imidazole and ultra-filtrating, protein concentration and purity were measured. The SDS-PAGE results have shown that the band at approximately 70 kDa is very clear (Figure 1D,E). The concentrations of proteins are high enough for this study.

### 2.2. Mutations in PLO Allow for Host Tolerance to rPLO

**Figure 2:**
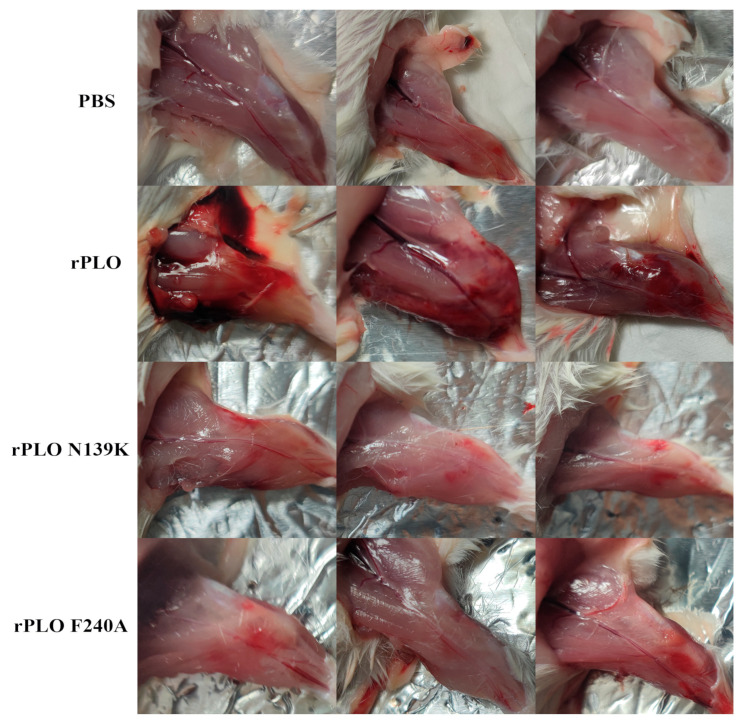
Effects of rPLO and its mutants on mouse muscle tissue injury.

Due to PLO being commonly considered as a bacteriocin of extracellular bacteria, we attempted to investigate the importance of PLO toxicity to *T. pyogenes* through in vivo infections. The muscle tissues of mice were severely damaged by rPLO with bleeding points, inflammatory tissue hardening, and tissue swelling (Figure 2). In contrast, there were no significant pathological changes observed in the tissues of mice injected with PBS, rPLO N139K, and rPLO F240A after the mice were humanitarianly sacrificed. This indicates that rPLO has a strong ability to cause inflammatory damage in mouse muscle tissue. The loss of the ability of rPLO with single-point mutations indicates that these two points play important roles in maintaining the function of PLO. Compared with mice infected with rPLO N139K and rPLO F240A, introducing rPLO into muscle tissues can induce high inflammatory responses.

### 2.3. Mutations Weaken the Disruption of rPLO on Tight Junctions and K^+^ Efflux

**Figure 3:**
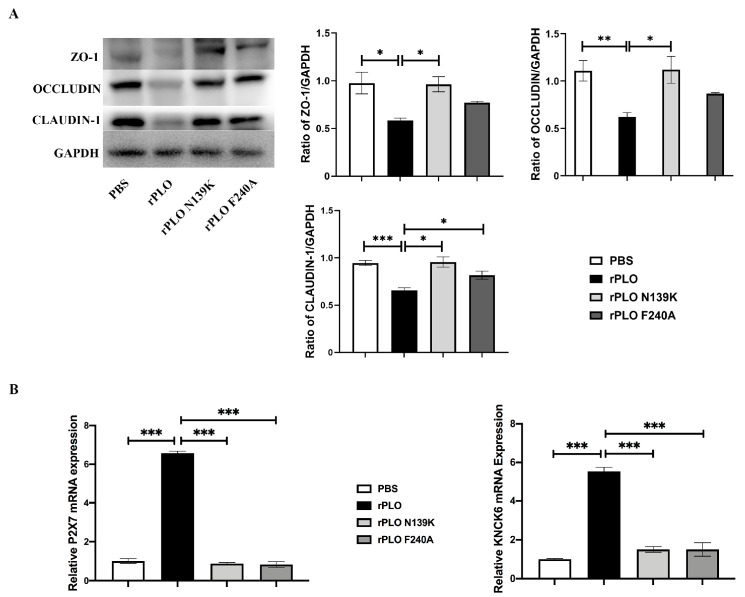
Effects of rPLO and its mutants on tight-junction proteins and K^+^ efflux channel genes. (**A**) The effect of rPLO and its mutants on tight-junction proteins in mouse muscle tissues; (**B**) The effect of rPLO and its mutants on the expression levels of P2X7 and KNCK6. Transcript levels were normalized to GAPDH. Data are shown as mean ± SEM from three independent replicates (* *p* < 0.05, ** *p* < 0.01, *** *p* < 0.001).

In order to investigate whether single-point mutations can alleviate the damage of tight-junction proteins induced by rPLO, thereby restricting K^+^ efflux, this study analyzed the expression levels of tight-junction proteins and K^+^ efflux-related genes in mouse muscle tissues (Figure 3). The expression levels of ZO-1, Occludin, and Claudin-1 in mouse muscle tissues infected by rPLO were significantly lower than that of the control group (Figure 3A). In contrast, compared with mice infected with rPLO, the expression levels of Occludin, Claudin-1, and ZO-1 were significantly increased in mice injected with rPLO N139K and rPLO F240A. Our results indicate that single-point mutations can significantly weaken the disruption of tight-junction proteins in mouse tissues, maintaining tissue integrity. By detecting genes related to the K^+^ efflux pathway, we found that rPLO can increase the gene expression levels of P2X7 and KNCK6 (Figure 3B). However, the proteins with two single-point mutations do not have the ability to activate genes related to K^+^ efflux. K^+^ efflux is also associated with the activation of the NLRP3 inflammatory pathway.

### 2.4. Mutations Alleviate the Activation of the NLRP3 Inflammatory Pathway

**Figure 4:**
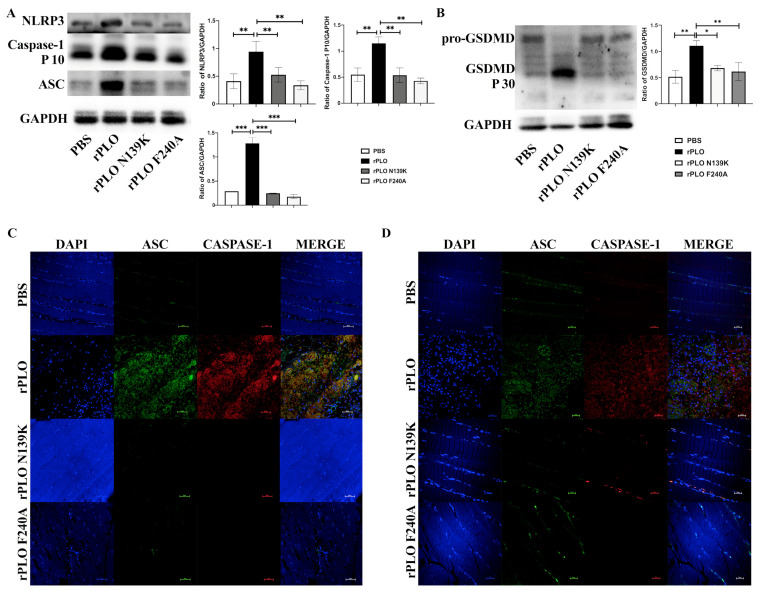
The effect of rPLO and its mutants on the expression levels of inflammation-related proteins in mouse muscle tissues. (**A**) Western blotting to detect different effects of rPLO and its mutants on NLRP3, ASC, and Caspase-1; (**B**) Western blotting to detect different effects of rPLO and its mutants on the pyroptosis-related protein GSDMD; (**C**,**D**) Immunofluorescence to detect the effects of rPLO and its mutants on ASC, Caspase-1 and NLRP3. Data are shown as mean ± SEM from three independent replicates (* *p* < 0.05, ** *p* < 0.01, *** *p* < 0.001).

Through the detection of the NLRP3-inflammatory-pathway-related proteins, we found that compared with the control group, NLRP3, ASC, and Caspase-1 were significantly activated in the tissues of rPLO infected mice (Figure 4A). The mutants did not significantly activate these proteins. Further detection of GSDMD revealed that rPLO also activated the pyroptosis-related protein (Figure 4B). Similarly, neither mutant showed significant signs of activating GSDMD, proving that rPLO N139K and rPLO F240A have basically lost their main function as the major bacteriocin secreted by *T. pyogenes*. To further validate our hypothesis, we also conducted immunofluorescence experiments of tissues (Figure 4C,D). In rPLO injected mouse tissues, ASC, NLRP3, and Caspase-1 were all activated, and their expressions were enhanced. This is consistent with our previous results, which also proves that single-point mutations cause irreversible clearance of PLO functions.

### 2.5. The Effect of K^+^ on rPLO Activation of the NLRP3 Inflammatory Pathway

**Figure 5:**
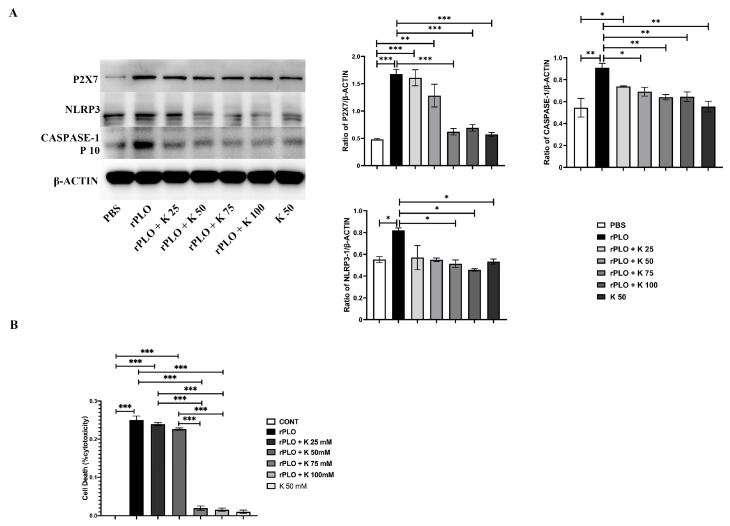
Mutants of rPLO inhibit K^+^ efflux and alleviate cellular inflammatory damage by reducing drilling ability. (**A**) Adding different concentrations of K^+^ reduces the expression levels of NLRP3-inflammatory-pathway-related proteins and P2X7 caused by rPLO; (**B**) Adding different concentrations of K^+^ reduces the release of cell LDH caused by rPLO. Data are shown as mean ± SEM from three independent replicates (* *p* < 0.05, ** *p* < 0.01, *** *p* < 0.001).

To investigate the mechanism by which rPLO activates the NLRP3 inflammatory pathway, different concentrations of K^+^ were added to endometrial epithelial cell culture medium. The results showed that the expression level of P2X7 was gradually downregulated with increasing K^+^ concentration (Figure 5A). Adding 25 mM K^+^ can inhibit the efflux of intracellular K^+^, thereby reducing the activation of NLRP3, ASC, and Caspase-1. In Figure 5B, the results of LDH indicate that adding K^+^ can effectively reduce the cytotoxicity caused by rPLO and significantly reduce the efflux of intracellular lactate dehydrogenase. This further indicates that PLO activates the NLRP3 inflammatory pathway by drilling holes in the cell membrane, which leads to the K^+^ efflux. Two mutants also successfully removed the pore-forming ability and inflammatory pathway activation ability of rPLO.

## 3. Discussion

Billington et al. cloned and sequenced the *plo* gene encoding the PLO toxin, which is located in the open reading frame (ORF) of the 1605 bp gene of *T. pyogenes* [13]. A common ribosome binding site, two promoter sequences, and three co-repeating sequences were found upstream of the *plo* gene, with a transcription termination region located downstream of the gene [14]. In addition, Rudnick et al. found that the *plo* gene, along with the aforementioned sequence and ORF (orf121), encodes an unknown functional protein of 13.4 KDa, forming a 2.7 kb gene island characterized by a decrease in G+C content (50.2%) [15,16]. On both sides of this gene island, there are two housekeeping genes *smc* and *ftsY* (62.5% G+C) located in the genes of *T. pyogenes* [15,17]. The difference between virulence island and flanking genes indicates the possibility of horizontal transfer of the *plo* gene, although no integrase or transposon sequences were found in this chromosomal region. The *plo* gene exists in all wild-type strains of *T. pyogenes*. 

According to observations, the in vitro hemolytic activity of *T. pyogenes* is in its growth phase, which depends on the peak from early growth to stable phase [18]. It indicates that *plo* is upregulated at this stage and a significant increase in *plo*-specific mRNA is indeed observed. Therefore, the hypothesis that PLO expression is controlled at the transcriptional level has been confirmed [18]. According to the *plo* gene sequence prediction, the length of the PLO molecule is 534 amino acids (aa); so, the molecular weight of the protein should be 57.9 kDa. Signal peptidase cleavage sites were found between 27 and 28 aa, indicating a mature PLO molecule of 55.1 kDa.

Currently, the methods for studying PLO are also very limited. We can predict the structure and function of PLO by studying other members of the CDC family. As a member of the CDC family, PLO exhibits cytotoxic effects on various host cells, such as red blood cells, polymorphonuclear neutrophils (PMNs), macrophages, epithelial cells, fibroblasts, and endometrial stromal cells [19,20,21]. The cell lysis activity of PLO is related to its ability to bind to the plasma membrane and form transmembrane pores. Some of CDC’s known secondary and tertiary structures exhibit significant similarities [22]. The crystal structure of PLO molecules may be homologous to the structure of other CDC molecules. Monomer PLO molecules are rich in β-folding elements, consisting of four structural domains (D1 to D4), where D2 and D3 are stacked together, with D1 located at the N-terminus and D4 located at the C-terminus of the molecule.

Domain 1 contains α-helix and β-folding element. The exact role of D1 in the pore formation process is still uncertain. Zhang et al. investigated the effect of replacing aspartic acid (D238) in this domain with arginine, indicating that D1 plays an important role in maintaining the pore-forming activity of PLO [20]. The investigation by Imaizumi et al. showed that Domain 1, especially the 55–74 amino acid region, is essential for the hemolytic activity of PLO [23]. Yan et al. also confirmed the importance of this region in Domain 1, demonstrating that each amino acid from 58 to 62 in PLO molecules is replaced, especially isoleucine 61, resulting in a complete loss of its hemolytic activity [24]. In this study, the structure of PLO indicates that the selected mutant residues 139 and 240 are located on the irregular coil of Domain 1, which is rarely studied among the four domains of PLO.

It seems that the main task of PLO’s cytolytic activity is to obtain free iron and other growth factors, which are crucial for bacterial replication in host cells [25]. In addition, the ability to lyse phagocytic cells protects bacteria from host immune responses. On the other hand, at lower concentrations, PLO can regulate host immune responses [26]. Although there have been some studies on the molecular structure and function of PLO, the exact role of this toxin in the pathogenesis of specific *T. pyogenes* infections (characterized by different disease courses and clinical manifestations) is still unclear. This study successfully selected two highly conserved sites of PLO for single-point mutations through comparative and in-depth analysis. Successfully expressing and purifying PLO and its mutations in vitro have laid the foundation for subsequent comparative studies on the structure and function of proteins of the CDC family.

Although numerous reports have confirmed that members of the CDC family can interact with host cells in various ways, innate membrane-binding ability may prompt those bacteriocins to preferentially adhere to the cell membrane [20,27]. Therefore, instead of using these toxins as ligands to study their characteristics, it is better to focus more on the adverse effects of their pore-forming ability on the host. Therefore, further research on the pore formation mechanism of these toxins has clinical application value. Our previous data indicated that a single-residue mutation in Asn139Lys in PLO terminated the pore-forming ability of PLO on the cell membrane, while the Phe240Ala mutation only retained a relatively limited pore-forming ability compared to the Asn139Lys mutant, indicating that the 139th residue Asn and 240th residue Phe are crucial for the pore-forming ability of PLO [7]. Essentially, the overall structure of these mutants remains unchanged compared to the overall structure of PLO monomers. Some research reports suggest that the loss of essential cation π interactions due to the replacement of residues positively correlated with the conformation of Domain 1 and Domain 3 is the main reason for the loss of pore-forming ability of Ply NH (another important member of the CDC family) [28]. Interestingly, these subtle substitutions completed by single-residue mutations are sufficient to effectively limit the function of PLO mutants. From an evolutionary perspective, this may be more beneficial as new features can appear in a short period of time without causing significant changes in protein structure, and the lack of pore-forming ability among CDC members may indirectly provide bacteria with the possibility of intracellular survival to evade antibacterial autophagy [28]. Therefore, further research on the impact of CDC protein dysfunction on bacteria will also provide impetus for studying the relationship between bacteria and hosts.

*T. pyogenes* typically causes inflammatory diseases of different species, including miscarriage, arthritis, endocarditis, mastitis, pneumonia, and osteomyelitis [5]. PLO, as the main weapon of *T. pyogenes*, performs destructive tasks by disrupting cellular barriers and evading host immune responses [23]. The results of mouse tissues indicate that rPLO activates the NLRP3 inflammatory pathway, causes significant tissue damage, and induces inflammatory cell infiltration into mouse tissues. Recent studies have shown that the activation of inflammatory cytokines is associated with potassium ion efflux, which can lead to the activation of the NLRP3 pathway in the NLR family [29,30,31,32]. More reports indicate that PLO can upregulate the levels of inflammatory cytokines in different cells [33,34]. However, compared with rPLO, the ability of rPLO F240A and rPLO N139K to activate the inflammatory pathway is significantly reduced, which may be related to their complete or partial loss of pore-forming ability to disrupt tight junctions leading to the K^+^ efflux. Moreover, compared to these mutants, rPLO is more easily recognized by the innate immune system of mice, thereby mobilizing immune cells. We speculate that this difference may be due to the essential changes in membrane-binding ability caused by the substitution of different amino acids.

## 4. Materials and Methods

### 4.1. Bacterial Strains, Plasmids, and Chemicals

The *Trueperella pyogenes* used in this study was a generous gift from Professor Zhu’s laboratory. The plasmids and chemicals used in this study were selected according to the previous methods [7,35,36]. Briefly, the *plo* gene and the single-point mutant *plo* genes were cloned into the pET32a plasmid (Addgene). Preparation of the single-point mutated genes was performed using the fusion PCR method. All chemicals and enzymes were purchased from Sigma Chemical Co. (St. Louis, MO, USA), Solaibio Life Sciences (Beijing, China), TransGen Biotech (Beijing, China), and Beyotime Biotechnology (Shanghai, China) unless otherwise specified. All sequencing work was completed by Sangon Biotech (Shanghai, China).

### 4.2. Generation and Purification of Toxin

The rPLO protein and mutants, rPLO N139K and rPLO F240A, were generated and purified as described previously [7]. Briefly, the fusion PCR product with correct sequencing was added to the Trans5α Chemically Competent Cell (Transgen Biotech, Beijing, China) with an amino-terminal His tag. After plasmid introduction, a single colony was selected and cultured for 12 h in 5 mL Luria Bertani (LB) Broth containing 100 mg/mL ampicillin at 37 °C for two generations. Bacterial solution and fresh LB Broth were mixed in a 1:100 ratio and incubated for 4–5 h until the measured OD_600_ was between 5 and 6. The isopropyl-L-thiogalactopyranoside (IPTG) inducer was added and incubated at 16 °C with a shaking speed of 120 rpm for 20 h. The final concentration of IPTG is 0.8 Mol/L. The bacterial solution was centrifuged at 8000× *g* for 10 min at 4 °C, then washed and re-suspended twice with pre-cooled PBS 10 mL, and sonicated with an ultrasonic crusher for 30 min. The supernatant and precipitate were centrifuged and collected at 8000× *g* for 10 min at 4 °C. Proteins were filtrated using ProteinIso^®^ nickel–nitrilotriacetic acid (Ni NTA, DP101, TransGen Biotech, Beijing, China) Resin filter column and eluted with different gradients of imidazole solutions. The protein concentration was measured by Nano Drop (ND-ONE-W, Thermo Fisher, Waltham, MA, USA), and the purity was then detected by sodium dodecyl sulfate-polyacrylamide gel electrophoresis (SDS-PAGE).

### 4.3. Biosecurity Statement

*T. pyogenes* was treated strictly in accordance with the State Council of the People’s Republic of China regulations on the biological safety of pathogen microbiology laboratories (000014349/2004-00195). All necessary, safe operations were conducted to avoid pathogen transmission and infection.

### 4.4. Animal Infection Experiment

A total of 24 CD1 mice (6–8 weeks) were randomly divided into 4 groups. The mice were injected intramuscularly with 300 μL PBS, rPLO, rPLO N139K, and rPLO F240A, with a total protein mass of 200 μg on days 1, 3, and 5, respectively. On the third day after the last injection, all mice were euthanized and muscle tissues from the injection sites were collected. The pathological changes in animal tissues caused by rPLO and rPLO mutants were compared using real-time quantitative PCR, Western blotting, and immunofluorescence.

### 4.5. Western Blotting

Mouse muscle tissues were taken for protein extraction. Muscle tissues in different groups were treated with radio-immunoprecipitation assay buffer (RIPA, Solarbio, Beijing, China) containing 1% phenylmethylsulfonyl fluoride (PMSF, Solarbio, Beijing, China) for 30 min for Western blotting. A total of 20 μg of protein was loaded to each well for SDS-PAGE, and the polyvinylidene fluoride (PVDF) membrane was activated by methanol (Sinopharm, Shanghai, China) for 1 min for transferring protein. The primary antibodies were rabbit polyclonal anti-ASC (1:500 dilution, 10500-1-AP), rabbit polyclonal anti-Claudin-1 (1:2000 dilution, 13050-1-AP), mouse monoclonal anti-ZO-1 (1:1000 dilution, 66452-1-Ig), rabbit polyclonal anti-NLRP3 (1:1000 dilution, 19771-1-AP), mouse monoclonal anti-GSDMD (1:5000 dilution, 66387-1-Ig) (ProteinTech Group, Rosemont, IL, USA), rabbit polyclonal anti-P2X7 (1:1000 dilution, PU358399S, Abmart, Shanghai, China), rabbit polyclonal anti-Caspase-1 (1:1000 dilution, ab179515), and rabbit polyclonal anti-Occludin (1:2000 dilution, ab216327) (Abcam) (Cell Signaling Technology, Danvers, MA, USA). The mouse anti-glyceraldehyde-3-phosphate dehydrogenase (GAPDH; 1:5000 dilution, 60004-1-Ig) and mouse anti-β-actin (1:5000 dilution, 66009-1-Ig) were applied for verifying the equal sample loading. The secondary antibodies included goat anti-mouse IgG (1:5000 dilution, SA00001-1) or goat anti-rabbit IgG (1:5000 dilution, SA00001-2) (ProteinTech Group).

### 4.6. Immunofluorescence

Different groups of mouse tissues were fixed in 10% formaldehyde solution. The samples were embedded with paraffin and sectioned for immunofluorescence experiments as described previously [37]. Briefly, primary antibodies NLRP3, ASC, and Caspase-1 were applied, and Alexa Fluor 488 goat anti-rabbit and Alexa Fluor 555 anti-rabbit were used as secondary antibodies. Nuclei were stained with 4′,6-diamidino-2-phenylindole (DAPI; Solarbio, Beijing, China). Glass coverslips were faced down on slides with the drip-proof anti-fluorescence quencher and mounted with neutral resin. The slides were observed and pictured with the Nikon A1 confocal laser scanning microscope with the same exposure.

### 4.7. Real-Time PCR

Mouse muscle total RNA of different groups was isolated using a Trizol reagent (Invitrogen, Carlsbad, CA, USA). The concentration of RNA was detected with a NanoDrop instrument (ThermoFisher, Waltham, MA, USA). The genomic DNA was removed before reversing transcription into first-strand complementary DNA (cDNA). The sequences of the primers used, which are P2X7 and KNCK6, are listed in Table 1. A no-template control and all the samples were used for the procedure which was performed in triplicate. The data of real-time PCR are exhibited with the method of the 2^−∆∆CT^ as fold-change.

### 4.8. Cytotoxicity Assay

According to the manufacturer’s instructions as described previously [7], the lactate dehydrogenase (LDH) release assay kit was used to measure the changes in the cytotoxicity of rPLO on endometrial epithelial cells under the premise of adding different concentrations of potassium ions.

### 4.9. Statistical Analysis

All data were performed and analyzed by GraphPad Prism 9 (GraphPad Software, Boston, MA, USA). The Western blotting results were performed by Image J software (Version 1.8.0, National Institutes of Health, Bethesda, MD, USA). All data analysis was performed with the statistical methods of one-way ANOVA. Multiple corrections were applied by Tukey’s honestly significant difference for a post hoc test. Data are presented as the mean ± SEM which were calculated from three similar parallel experiments. A *p* < 0.05 was considered statistically significant.

## 5. Conclusions

This study investigated the mechanism of PLO-induced inflammation in mouse muscle tissue by detecting unit point mutations in PLO. Our data also provide a theoretical basis and practical significance for future research on toxins and bacteria. PLO can activate the NLRP3 pathway and mediate the GSDMD cleavage through the destruction of the cell membrane and the efflux of intracellular K^+^. Our work partially explains why PLO exhibits pro-inflammatory properties. Single-point mutations of highly conserved amino acids in the hemolysin of *T. pyogenes* can significantly reduce the ability of PLO to cause inflammatory damage. These mutations may not be tolerated for hemolysin for all CDC family members.

## Figures and Tables

**Table 1:** Sequences of oligonucleotide primers used for real-time PCR.

Primer Name	Direction	Sequence (5′-3′)
P2X7	F	AGCCTGTTATCAGCTCCGTG
	R	CCTAACTTCGTCACCCCACC
KNCK6	F	CCTGGATGCCTTCGTGGAG
	R	AAGCGTGCTGGCGAAGAA

## Data Availability

All experimental data are included in this paper.
